# Infusing Oral Health Care into Nursing Curriculum: Addressing Preventive Health in Aging and Disability

**DOI:** 10.1155/2012/157874

**Published:** 2012-04-26

**Authors:** Joan Earle Hahn, Leah FitzGerald, Young Kee Markham, Paul Glassman, Nancy Guenther

**Affiliations:** ^1^Department of Nursing, College of Health and Human Services, University of New Hampshire, 4 Library Way, Hewitt Hall Room 279, Durham, NH 03824-3563, USA; ^2^UCLA School of Nursing, Factor Building, P.O. Box 956919, Los Angeles, CA 90095-6919, USA; ^3^University of the Pacific School of Dentistry, 2155 Webster Street, San Francisco, CA 94115, USA; ^4^Living Healthy with a Disability Program, California Department of Public Health, 1616 Capitol Avenue, Suite 74-660, P.O. Box 997377, MS 7214, Sacramento, CA 95899, USA

## Abstract

Access to oral health care is essential for promoting and maintaining overall health and well-being, yet oral health disparities exist among vulnerable and underserved populations. While nurses make up the largest portion of the health care work force, educational preparation to address oral health needs of elders and persons with disabilities is limited across nursing curricula. This descriptive study reports on the interdisciplinary development, implementation, and testing of an oral health module that was included and infused into a graduate nursing curriculum in a three-phase plan. Phase 1 includes evaluation of a lecture presented to eight gerontological nurse practitioner (GNP) students. Phase 2 includes evaluation of GNP students' perceptions of learning, skills, and confidence following a one-time 8-hour practicum infused into 80 required practicum hours. The evaluation data show promise in preparing nurse practitioner students to assess and address preventive oral health needs of persons aging with disabilities such that further infusion and inclusion in a course for nurse practitioners across five specialties will implemented and tested in Phase 3.

## 1. Introduction/Background

 Health disparities for intellectual and developmental disabilities (I/DD) often exist, including poorer health, unmet health needs, and problematic access to primary and preventive health services [1–6] including preventive oral health services and severe oral health disparities. The safety net to offset these disparities in oral health is lacking in a comprehensive system of care [7].

Access to oral health care is essential to promoting and maintaining overall health and well-being, yet only half of the United States population visits a dentist each year. Older adults and disabled individuals uniformly confront access barriers, regardless of their financial resources. The consequences of these disparities in access to oral health care can be associated with a number of conditions, including malnutrition, infections, diabetes, heart disease, and premature births. For example, periodontal disease is associated with diabetes and its sequelae, including stroke, transient ischemic attack, myocardial infarction, and intermittent claudication [8, 9]. In addition, various side effects of medications increase the risk of oral health disease (e.g., drug-induced xerostomia and gingival hyperplasia).

 The surgeon general reports that persons with disabilities (PWDs) have more dental disease, more missing teeth, and more difficulty obtaining dental care than other members of the general population [10]. Annually, 36.5 percent of severely disabled persons 15 years and older report a dental visit, compared with 53.4 percent of those with no disability [11]. Few states cover dental services for adults under Medicaid, and clinical preventive services are generally lacking for PWD [12]. Even in states with Medicaid coverage, low reimbursement rates and the reluctance of practitioners to accept those rates reduce the availability of care required for treating patients with disabilities [13]. Seniors face similar oral health disparities [14]. Of particular concern for nursing and oral health professionals is the fact that there is increasing evidence of the association of dental disease with general health conditions.

In July 2011, the Institute of Medicine (IOM) released a report, “*Improving access to oral health care for vulnerable and underserved populations”* [14], which examines the scope and consequences of inadequate access to oral health services in the USA. The IOM report supports the creation of a diverse workforce that is competent, compensated, and authorized to serve vulnerable and underserved populations across the life cycle. The report recommends ways to combat the economic, structural, geographic, and cultural factors that prevent access to regular quality care and recommends changes to incorporate oral health care into overall health care. One recommendation includes expanding the oral health work force by training physicians, nurses, and other nondental professionals to recognize risk for oral diseases. The report also suggests changing funding and reimbursement for dental care and adding or changing recommendations to revamp regulatory, educational, and administrative practices [14].

 Nurses make up the largest portion of health care persons in the work force today [15]. Nurses, nurse practitioners (NPs), and health educators are far more likely to encounter underserved and vulnerable populations than dental professionals, particularly family health and community nurses [16, 17]. Increasing nurses' awareness and knowledge about oral health in general can increase nurses' knowledge and skills in oral health care.

 Proper education and tools to assess oral health risk can be used in conjunction with other risk assessments to help guide early detection and prevention of oral disease [18]. Through individual and population-based screenings, nurses and NPs can identify at-risk individuals and assess and manage their oral health needs as well as make referrals to dental professionals when necessary. For example, NP comprehensive assessment led to the referral of 38.6% (*n* = 27) of 70 adults with intellectual and developmental disabilities for oral health counseling, based on identified unmet oral health needs [19]. Having the ability to identify potential health risk factors such as lifestyle, ethnicity, health status, and social determinants associated with oral health status risk, nurses can take an active role in health screening to discover any need for clinical preventive services, including dental preventive services, and can detect health problems for PWD [20]. Therefore, it is essential that these health care providers are familiar with the various risk factors to manage oral care and make appropriate referrals and intervention decisions as recommended by the IOM report.

Attention to oral health, including oral assessment and awareness of the negative consequences due to poor oral care, has been found lacking in nursing curricula [21], especially concerning oral health and elders [22]. In addition, a void exists in the literature on NP education about oral health and PWD [23], and general education of nurses about health promotion for PWD is deficient. In a national survey of 1,000 basic nursing education programs, over half of the schools reported little curriculum content regarding health promotion for PWD; this was attributed to lack of faculty time, interest, and expertise [24]. Innovative projects led by faculty and others can enhance the integration of new curriculum in nursing programs, including discipline-specific and interdisciplinary training. A small number of programs demonstrate collaboration between nurses and oral health professionals [25, 26] with overlapping competencies related to oral health [18].

 Nursing education that combines course work with “hands-on” experience has positively affected the attitudes of nursing students toward PWD [27]. High satisfaction with an educational opportunity that paired dental hygiene students with student nurses was reported by nursing students who completed training and clinical practicum performing oral assessments of school children [28]. An “infusion” or integration approach to curricular change that embeds new content about disabilities into existing health professional education is gaining attention and can address identified curricula needs.

 The specific aim of this project was to develop, test, and integrate an oral health module targeted toward oral health of elders and persons with disabilities into graduate nursing curriculum as a sustained learning activity for NP students. The long-term aim, by increasing NP's awareness and skills, is to promote health and prevent comorbid conditions among persons with disabilities. We will describe the development, implementation, and evaluation of the oral health module as well as how these activities, in a three-phase process, led to *inclusion and infusion* into existing nursing curriculum for graduate NP students (see Table 1 for activities according to phases).

## 2. Methods and Materials

### 2.1. Phase 1: Module Development

An initiative within a Cooperative Agreement with the Centers for Disease Control and the California Department of Public Health provided funds to develop curriculum and training materials for nursing students and other health care professionals. The California Department of Public Health, Safe and Active Communities Branch (SACB), Living Healthy with a Disability Program was awarded funding for “*Module E: Training of Professionals and Paraprofessionals,”* and UCLA was funded for a curricular project, entitled* “Disability Inclusion and Infusion in Nursing Education.” *The overall aim was to increase knowledge, skill, and confidence of nursing faculty, nursing students, oral health professionals, and other allied health care professionals in implementing effective health promotion and wellness strategies and interventions to promote health and prevent development of secondary conditions experienced by PWD. The University of California Los Angeles (UCLA) subcontracted with the Pacific Center for Special Care at the University of the Pacific School of Dentistry to develop oral health components. The curricular project was approved by the UCLA School of Nursing's faculty curriculum committee, the UCLA Institutional Review Board (IRB), and the Committee for the Protection of Human Subjects (CPHS), which is the IRB for the California Health and Human Services Agency.

 One nursing faculty member from UCLA School of Nursing, two dental faculty and dental staff from the University of the Pacific School of Dentistry, and the project manager from the California funding agency developed the oral health module via monthly teleconferences. The module has three main components, a didactic lecture, a clinical practicum, and an evaluation component. The components are outlined in Table 2.

### 2.2. Phase 1: Oral Health Lecture

 A dental faculty from UCLA School of Dentistry and a UCLA School of Nursing (SON) faculty worked in collaboration. The dental faculty member conducted a 120-minute lecture on oral health and elders that included a demonstration on how to conduct an oral health screening; students performed a return-demonstration using other students as patients. Students received written materials on oral health care for elders and oral heath for PWD, and dental and SON faculty reviewed content, respectively. For a list of content covered in the lecture, see Table 3. The lecture was provided to eight gerontology nurse practitioner (GNP) students during their first year master's level gerontological nursing theory course. The SON faculty member coordinated a satisfaction evaluation questionnaire at the end of the lecture.

### 2.3. Phase 1: Module Practicum Forms

 The practicum consisted of three forms for GNP students to use during the practicum. A description of the three forms follows.

 The Dental Assessment for the Nondentist was developed originally for case managers and nondental laypeople. Essentially, this form provides an instruction sheet to guide assessment using a series of questions and decision-making based on two protocols. The first protocol guides the decision for dental follow-up as routine, emergent, or urgent. The second protocol guides the decision about recommendations for help or accommodations for the PWD on oral preventive education and if oral health training is indicated for a caregiver. The second form, the *Oral Health-Screening Exam Results,* provides a document to record oral health assessment results and possible treatment needs. This includes a checklist to note recommendations for oral health providers based on the client's abilities to engage in the oral health assessment. The third form was the *Oral Health Prevention Plan*, which is used to record a preventive oral health plan encompassing, as needed, documentation of physical skills, a behavioral plan, special aids, denture care, other preventive actions (e.g., fluoride rinse, and record of dental visits, location), and special considerations for follow-up. A copy of these forms can be found in Supplementary Material available online at doi:10.1155/2012/157874.

### 2.4. Phase 1: Identification of Clinical Practicum Site

 In Phase 1, we collaborated with a local case management/service coordination agency for persons with intellectual and developmental disabilities to arrange the contractual paperwork to establish this agency as a nursing clinical practicum site. The nursing faculty member had pilot tested a multidimensional health screening using screening tools commonly administered in a comprehensive geriatric assessment that have been pilot tested with adults with I/DD [19]. A social worker or service coordinator recruited persons aging with lifelong developmental disabilities to attend the health fair.

### 2.5. Phase 2: Module Integration

 In Phase 2, we began the first infusion of the oral health module into nursing curricula. We infused the oral health practicum into the existing multidimensional health screening held for persons aging with I/DD at the local case management agency located within five miles of the school. The first practicum occurred with a cohort of first-year GNP students who had attended the lecture. The oral health module was included in the clinical practicum activities of this GNP clinical group during a clinical theory/practicum course offered during their second year.

 A SON GNP clinical faculty member at the health fair supervised the cohorts of GNP students. At each fair, two students formed a team to interview and assess one client. Following the screening, each client and/or caregiver received education on pertinent health issues. Arrangements were facilitated for clients with immediate health concerns, with either a health care provider or, if needed, at the local hospital emergency department. The enthusiasm of the agency about this combined oral health and multidimensional health-screening fair led to a yearly event called a Geriatric Health Screening Fair. Thus, this full-day (8-hour) practicum for GNP students was held as a part of a health fair for the geriatric client with developmental disabilities. GNP students received 8 hours of credit toward the 80 clinical hours required in this second year course.

 Over the course of Phase 2, 29 GNP students and 53 clients took part in four Geriatric Health Fairs (practica) from 2008 to 2011. Each GNP student received the resource information packets on oral health care and geriatric screening for elders and PWDs prior to the practicum. Each year, prior to the practicum, the SON GNP faculty member provided and reviewed guidelines for conducting an oral health assessment and using the three forms. At this point in the program, GNP students had been taught and had practiced basic oral health assessment. The comprehensive health-screening packet included geriatric screening tools as well as forms for the oral health screening practicum to allow for an experience with a comprehensive multidimensional screening.

### 2.6. Phase 2: Module Practicum Evaluation

 We developed two practicum evaluation tools. The practicum included an evaluation questionnaire for the GNP students and a tool tailored for persons with intellectual and developmental disabilities. The evaluation tool for GNP students includes Likert-type scale questions using a 5-item scale as well as some open-ended questions. The tool for persons with I/DD uses a 4-item Likert Scale with smiley faces to assess their level of satisfaction from “highly satisfied (pleased)” to “not at all satisfied” in four areas: materials received, accommodations, health education, and visit with the nurse.

## 3. Results

 Data were collected between 2008 and 2011. The lecture was given to eight first-year master's level gerontological NP students in 2008. Only one set of lecture evaluation data is available as, subsequently, the stand-alone lecture was replaced with integrated content within the SON clinical faculty member's clinical conferences with the students as the practicum was infused into this clinical section's activities.

### 3.1. Participants

The GNP students who attended the lecture were first-year graduate nursing students specializing in adult/gerontological nursing. The GNP students, who attended the practicum, were in their second year. The 53 persons with I/DD who volunteered to attend the health fair were adult clients of a local service coordination agency. Most of the clients were aged 55 and older. Each client met the criteria as having a “developmental disability” according to the state of California's definition. The majority had some level of intellectual disability in the moderate or mild range and were ambulatory and able to communicate in words. However, etiologies and diagnoses associated with having a developmental disability were unknown and not collected as data.

### 3.2. Lecture Evaluation

 The oral health module lecture evaluation had a 62.5% response rate from the eight GNP students who attended the lecture. The lecture was well received with 100% agreement on a rating of “5” or “liked a lot” on all of the items. There was 100% student agreement on learner gain at the highest rating (5: an abundance of information) for topics: preventive oral health care and the role of the advanced practice nurse (APN) in providing preventive oral health care for elders and for persons with disabilities. See Table 4 for mean levels of reported GNP student satisfaction and their estimates of learner gain.

### 3.3. Practicum Evaluation: GNP Students

 A total of 23 GNP students filled out an evaluation at the completion of the oral health practicum (held between 2008 and 2011). The survey response rate is 79.3%. In terms of overall satisfaction with the practicum, 100% expressed “good” to “excellent” satisfaction. Over 70% rated “good” to “excellent” having improved confidence (72.8%) and skills (77.3%) in working with PWD. Additionally, overall satisfaction with the oral health component of the practicum was 72.9% (see Table 5 for mean ratings).

 Anecdotal comments by GNP students showed the value of the experience and the desire of NP students to have this content in their coursework (see Table 6 for anecdotal NP student comments arranged by theme). Comments from GNP students suggesting improvements were related to wanting to have more time for the practicum or having more educational lecture materials about PWD prior to the practicum.

A comment from the SON clinical instructor who supervised the GNP students indicated the ability of students to gain skills in working with elders with disabilities. The clinical experience gave them (*the GNP students*) an eye opening experience and motivation to become an effective negotiator to enhance cooperation with oral exam and oral care, especially with those who would display anxiety lending to minimal cooperation with the oral examination.

### 3.4. Practicum Evaluation: Elders with Disabilities

**Figure 1:**
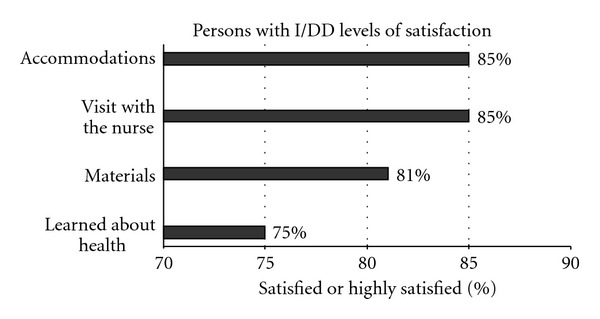
Rating of satisfaction by persons with I/DD with geriatric and oral health screening.

 Evaluation data were collected from 26 older adults seen by GNP students (49.1% response rate). Seventy-five percent or more reported being satisfied or highly satisfied on four items with the highest ratings for visit with the nurse and the accommodations that were made during the exam (see Figure 1 for rating of satisfaction by persons with I/DD).

 Another finding that shows the value of NP students doing the evaluation is that the majority of the individuals with I/DD who were seen by the GNP students during practice were referred for follow-up support for an identified health problem. To give an indication of the ability of nurses to recognize issues and the complexity of health issues of adults with I/DD, we present the specific findings from one of the health screenings (see Table 7 for the types of referrals made for the 16 individuals who were seen by the GNP students). There were only three clients who had no need for a referral. The referral to a dental provider was the second most frequent type of referral.

## 4. Discussion

 This appears to be the first reported educational intervention study that included infused, integrated, and reinforced oral health content and skills within gerontological curriculum and courses for GNP students. The project that led to the development and testing of an oral health module with didactic and practicum experiences for advanced practice nursing students was integrated into a theory-based gerontology course and a practicum for GNP students. This allowed for a clinical experience in conducting an overall comprehensive health screening with older adults with disabilities inclusive of oral health. For this eight-hour practicum, the students received credit toward their overall credit hours of clinical time. The majority of GNP students and the majority of PWD perceived the practicum experience as a whole, and specifically on oral health care, very favorably. Educated to provide safe, efficient, patient-centered quality, and equitable care, nurses can promote a comprehensive approach to health care, emphasizing the overall health and wellness of the patient, including oral health. Students were able to identify the need of PWD to get follow-up dental care as well as other health professional follow-up including follow-up nursing services from the case management agency. This opportunity not only provided the opportunity to gain a mastery of NP skills in assessment, communication, planning, and health promotion, but also it gave the nurses experience with both the older adult and the adult with disabilities. This was a logical infusion into content on conducting comprehensive geriatric screening to promote health and well-being among elders, in this instance, adults aging with disabilities, who are often underserved.

 The literature cites the challenges of integrating new curriculum into existing nursing curricula. Our project mirrors the finding that infusion and inclusion or integration of new content into nursing curricula takes time with a need for ongoing nurturing and support during the integration phase [29], which in this case was over four to five years. In addition, it takes champions. At each of the three phases, one nursing faculty member played a key role in the process. What facilitated this experience was the principle investigator's extensive experience with PWD, the interest of two other nursing faculty members who valued the importance and had willingness to take leadership to infuse and include the module into their courses.

 Other positive influences were the funding that gave credibility to the initiative and provided the opportunity for multidisciplinary collaboration among faculty, the endorsement of the curricular project by School nursing faculty, and the willingness of a clinical faculty member to participate and to add and sustain this clinical experience as part of clinical hours for students. Establishing a clinical contract for the agency as a new clinical site facilitated continued student opportunities at this site. We found that the practicum portion of the module has continued annually, in part due to two champions: the first, a faculty member who has taken a group of students each year to a regional center site, and the second, a clinical regional center staff liaison who assures that the event continues through recruitment of interested PWD. These partners are vital in coordinating and continuing this practicum for students.

### 4.1. Limitations

 Limitations of this study are the small numbers of students and the lack of a control group. The student group was targeted to GNP students who were part of only one clinical group of NPs across the nursing program. These limit the generalizability of the findings. We lacked a rigorous assessment of learner gain. A pre/posttest of knowledge, skills, and attitudes would add credibility that the intervention was associated with these gains and will be implemented in the next phase of this project. Further dissemination and testing are warranted in both undergraduate and graduate nursing programs.

### 4.2. Future Direction

 The next phase of the infusion and inclusion of the oral health module for elders and persons with disabilities will be for a cohort of 90 NP students who will enroll next quarter in a second year NP course that combines classes for theory content and clinical hours. Students will receive all components of the oral health module: 2-hour lecture, practicum with the three oral health forms, and student evaluation tools for lecture and practicum. This phase will add a pre/posttest for evaluation of learner gain. In addition, we will infuse the oral health practicum activities into the ongoing clinical practicum activities at the assigned clinical sites for each student. Each NP student will be required to conduct a minimum of 12 oral health screening assessments as part of their clinical NP practicum at sites where they are assigned (approximately one per week). Students will upload each oral health assessment into an internet-based course management system titled Modular-Object-Oriented Dynamic Learning Environment (Moodle). Oral health assessments and preventive oral health plans for clients seen will be discussed with clinical faculty at each postclinical conference session. Seven clinical faculty members will be involved. This cohort of NP students will include the NP specialty areas of acute care, adult/gerontological, adult/occupational health, family, and oncology nursing. Future research includes long-term follow-up evaluation to evaluate the sustained impact of this educational intervention on the NP's routine use of oral health screening and preventive education in postgraduation practice.

## 5. Conclusion

This innovative educational intervention study met with success and showed the receptiveness of the students to receiving knowledge and practicing skills in an area of nursing that may get overlooked or not emphasized enough, given its critical nature to health, and the lack of experience, especially to work with clients with disabilities. PWD and elders face oral health disparities. In accordance with the 2010 IOM Future of Nursing report, nurses are poised to help bridge the gap between coverage and access, to coordinate increasingly complex care for a wide range of patients and to fulfill their potential as primary care providers to the full extent of their education and training [30].

Evidence supports that nurses, once better educated and engaged, can provide safe, effective primary care services leading to improved health of the nation in a variety of settings. Utilizing nurses and NPs to their full potential, expanding their knowledge of oral health, we can meet the diverse needs of the public, including the oral health needs of this underserved and vulnerable population. The next steps of this project are to disseminate the Oral Health Module more widely and to evaluate the outcomes of nurses in promoting good oral health among not only PWD, but for all Americans.

## Figures and Tables

**Table:** (a) Phase 1: curriculum development and testing

Project phase	Activities	Methods	Participants	Data and response rate	Outcomes of activities
	Development of oral health forms and evaluation methods Identification of resources (for elders and PWDs)	Curriculum development with collaboration of dental and nursing faculty/staff and project director	Dental faculty/staff (2) Nursing faculty (1) Project Director (1)		Resource list Lecture evaluation tool Oral health forms (3) Practicum evaluation tool
Module development and testing	2-hour didactic lecture with oral health screening demonstration and GNP student return demonstration and post intervention evaluation	Educational intervention with postlecture evaluation	Nurse faculty member (1) Dental faculty member (1) GNP students (8)	Lecture Evaluation Tool GNP students *N* = 8 62.5%	High level of reported student satisfaction with module and learning of content
	Practicum site coordination Collation of educational and screening packets Recruitment of faculty and students		Nursing faculty member (1) Clinical nursing faculty (1)		Agency established as clinical practicum site Established an annual health fair conducted by NP students

PWDs: persons with disabilities; GNP: gerontological nurse practitioner.

**Table:** (b) Phase 2: module integration (clinical section)

Project phase	Activities	Methods	Participants	Data and response rate	Outcomes of activities
Infusion and inclusion into one clinical section	Integration of oral health content and resources in GNP clinical practicum section Practicum held at Geriatric Health Fair with oral and multidimensional health screenings for elders with I/DD	Practicum educational intervention with postlecture evaluation for GNP students and for elders with I/DD who attended the health fair	Clinical nursing faculty (1) Agency staff liaison (1) GNP students (23) PWDs (45)	Practicum evaluation by GNP students *N* = 23 79.3% *Elders with I/DD * *N* = 45 49.1%	Favorable ratings by GNP students on learning, improvement in confidence and skills and satisfaction with materials, tools, and practicum Favorable satisfaction ratings by elders with disabilities on help and visit by nurse, materials and learning about health

GNP: gerontological nurse practitioner; I/DD: intellectual and/or developmental disabilities.

**Table:** (c) Phase 3: module integration (nursing program)

Project phase	Activities	Methods	Participants	Data and response rate	Outcomes of activities
Integration into one NP course in graduate nursing program^a^	Integration of oral health module (lecture, practicum, and evaluation tools) into one course for all NP students in graduate nursing program activities	Educational intervention: 2-hour lecture by dental faculty, 12 required oral health screenings and preventive oral health plans per each NP student in practicum, and pre/postevaluation	Dental faculty member (1) Nursing faculty/Course coordinator (1) Nursing clinical faculty (7)^c^ NP students (90) Clients (practicum) = ~1080	Forthcoming in next academic quarter	Learner gain based on pre/posttest Learner satisfaction, confidence and skill development

NP: nurse practitioner.

^a^Course is inclusive of NP students in acute care, adult/gerontology, family, adult/occupational health, and oncology graduate master's NP program. ^b^All NP students in program except for pediatric NP students. ^c^One clinical faculty member per section of twelve students.

**Table 2:** Components of oral health module.

(i) Classroom lecture
(a) Taught with collaboration of nursing and dental faculty
(b) Didactic lecture with photos
(c) Demonstration of oral screening by dental faculty member
(d) Return demonstration by students
(e) Packet of resource materials on oral health for elders and PWDs

(ii) Practicum with persons aging with disabilities
(a) Packet of resource and teaching materials shared with students
(b) Forms for use in practicum
(1) Dental assessment for the nondentist
(1a) Assessment instruction sheet and protocols to guide recommendations
(2) Oral health-screening exam results
(2a) An oral health assessment form to record findings, treatment needs, and recommendations for oral health
(3) Oral health prevention plan
(3a) Identifies plan for overcoming obstacles to dental health

(iii) Module evaluation
(a) Lecture evaluation
(1) Likert-type scale survey with open-ended questions
(2) Rating of learning and improved confidence and skills
(b) Practicum evaluation
(1) For nursing students
(2) For persons with disabilities

**Table:** (a)

*Elders* ^ a^
Oral health screening
Oral health screening procedures
Age-associated changes
Non-age related conditions
Oral cancer
Xerostomia
Tooth decay/loss
Periodontal disease
Immune-related disease
Recognizing common oral health conditions for elders
(with photos and descriptions)
(e.g., candida, keratosis, “hairy leukoplasia” squamous cell carcinoma, ulcers, nicotine stomatitis)
Oral health prevention
Need for immediate dental treatment
Oral hygiene
Denture fit and denture care

^a^The lecture content outline on elders is from the lecture presented by S. Spackman to gerontological nurse practitioner students. The lecture has elements from curricular content developed by J. Bauer and S. Spackman for the program in Geriatric Dentistry at UCLA School of Dentistry, Los Angeles, USA.

**Table:** (b)

*Persons with intellectual and developmental disabilities* ^ b^
Definitions of disability
Demographics and oral health disparities among persons with I/DD
Conditions associated with developmental disabilities
(e.g., intellectual disability, Down syndrome, cerebral palsy, Fragile X syndrome)
Common oral health conditions
(e.g., cheilitis, xerostomia, malocclusion)
Other oral health concerns
(e.g., bruxism, pica, GERD, pocketing food)
Review of oral health assessment and preventive oral health education and practice
Urgent, emergent, routine follow-up
Positioning, accommodations, and adaptive equipment
Consideration of fears or anxiety related to oral care
Roles and teaching of caregivers

^b^The lecture content outline about oral health and persons with intellectual and developmental disabilities was developed from a number of resources including *Developmental Disabilities and Oral Health: *Strategies for Providing Oral Care to People With Developmental Disabilities—Practical Oral Care for People with Developmental Disabilities Series For Health Professionals—available at http://www.nidcr.nih.gov/OralHealth/Topics/DevelopmentalDisabilities/.

**Table 4:** Gerontological nurse practitioner evaluation of oral health module lecture (*n* = 5).

Category item	*M*
*Learning content* ^ a^	
Preventive oral health care	5
Role of APN in preventive oral health care for elders	5
Role of preventive oral health care for PWDs	5
*Satisfaction* ^ b^	
Materials on preventive oral health care	5
Overall rating of didactic	5
Overall rating of demonstration	5
Overall rating of return demonstration portion	5

^a^Based on Likert scale (1: *none* to 5: *learned an abundance of information*).  ^b^Based on Likert scale (1: *not satisfied at all* to 5: *highly satisfied*).

APN: Advanced Practice Nurse; PWDs: persons with disabilities.

**Table 5:** Gerontological nurse practitioner evaluation of practicum using evaluation tool.

Category item	*M* (SD)
*Learning* ^ a^	
Conducting a geriatric health screening with PWDs	3.65 (0.94)
Identifying need for preventive education or support	3.48 (0.90)
Identifying need for routine or emergent/urgent follow-up	2.91 (1.16)
*Confidence and Skills* ^ b^	
Rating of improved confidence	3.18 (0.85)
Rating of improved skills	3.23 (0.81)
*Satisfaction* ^ b^	
Overall rating of oral health components	3.27 (1.08)
Rating of Dental Assessment for the Nondentist form	3.32 (1.09)
Overall rating of practicum	3.86 (0.77)

^a^Based on Likert scale (1: *not much* to 5: *an abundance of information*).^b^Based on Likert scale (1: *poor* to 5: *excellent*);

PWDs: persons with disabilities.

**Table 6:** Gerontological nurse practitioner anecdotal comments about practicum.

Theme/category
Comments
Oral health
Oral health component was great!
(liked) guided assessment
Materials and Setting
(liked) the screening tools, availability of space and instruments
Need for Training
Is often overlooked area in this patient population
I enjoyed learning how we can be an advocate for clients, families, and caregivers
This was an enlightening experience.
Great experience! Wish we had more time here.
Attitude
Excellent clinical rotation and experience. I feel more at ease/comfortable with assessing this population.

**Table 7:** Types of referrals made by gerontological nurse practitioner students following a geriatric and oral health screening in 2008 (*N* = 16).

Referral type	*n*	%
Primary care provider	6	37.5
Dentist	3	18.8
Physical therapist	3	18.8
Nurse^a^	2	12.5
Ophthalmologist	1	6.3
Psychiatrist	1	6.3
Service coordinator^a^	1	6.3

^a^These personnel were from the service coordination agency.
